# Functional diversity in the color vision of cichlid fishes

**DOI:** 10.1186/1741-7007-8-133

**Published:** 2010-10-28

**Authors:** Shai Sabbah, Raico Lamela Laria, Suzanne M Gray, Craig W Hawryshyn

**Affiliations:** 1 Department of Biology, Queen's University, Kingston, ON, K7L 3N6, Canada; 2 Department of Biology, McGill University, Montreal, QC, H3A 1B1, Canada; 3 Centre for Neuroscience Studies, Queen's University, Kingston, ON, K7L 3N6, Canada

## Abstract

**Background:**

Color vision plays a critical role in visual behavior. An animal's capacity for color vision rests on the presence of differentially sensitive cone photoreceptors. Spectral sensitivity is a measure of the visual responsiveness of these cones at different light wavelengths. Four classes of cone pigments have been identified in vertebrates, but in teleost fishes, opsin genes have undergone gene duplication events and thus can produce a larger number of spectrally distinct cone pigments. In this study, we examine the question of large-scale variation in color vision with respect to individual, sex and species that may result from differential expression of cone pigments. Cichlid fishes are an excellent model system for examining variation in spectral sensitivity because they have seven distinct cone opsin genes that are differentially expressed.

**Results:**

To examine the variation in the number of cones that participate in cichlid spectral sensitivity, we used whole organism electrophysiology, opsin gene expression and empirical modeling. Examination of over 100 spectral sensitivity curves from 34 individuals of three species revealed that (1) spectral sensitivity of individual cichlids was based on different subsets of four or five cone pigments, (2) spectral sensitivity was shaped by multiple cone interactions and (3) spectral sensitivity differed between species and correlated with foraging mode and the spectral reflectance of conspecifics. Our data also suggest that there may be significant differences in opsin gene expression between the sexes.

**Conclusions:**

Our study describes complex opponent and nonopponent cone interactions that represent the requisite neural processing for color vision. We present the first comprehensive evidence for pentachromatic color vision in vertebrates, which offers the potential for extraordinary spectral discrimination capabilities. We show that opsin gene expression in cichlids, and possibly also spectral sensitivity, may be sex-dependent. We argue that females and males sample their visual environment differently, providing a neural basis for sexually dimorphic visual behaviour. The diversification of spectral sensitivity likely contributes to sensory adaptations that enhance the contrast of transparent prey and the detection of optical signals from conspecifics, suggesting a role for both natural and sexual selection in tuning color vision.

## Background

Vision is central to the survival of animals. Visual cues are used for orientation, detecting prey, avoiding predators and communication. The visual process starts with visual pigments absorbing light and initiating a photochemical cascade that leads to neural signaling, perception and ultimately visually mediated behavior. A common method for studying vision is the measurement of spectral sensitivity. Spectral sensitivity is the relative efficiency of detection of light as a function of wavelength. Spectral sensitivity is used to describe the characteristics of visual pigments found in cone photoreceptors in the retina, and it is particularly useful in describing the mechanisms of color vision [1,2].

Comparative studies in vertebrates established the presence of four spectrally distinct classes of cone visual pigments produced by several different opsin genes: *SWS1 *being the ultraviolet/violet-sensitive class (355-440 nm), *SWS2 *being the short wavelength-sensitive class (410-490 nm), *RH2 *being the mid-wavelength-sensitive class (470-530 nm) and *LWS *being the long-wavelength-sensitive class (495-570 nm) [3,4]. These opsin genes have been identified in the earliest vertebrate lineage, the jawless fishes or Agnathans (lamprey) [5], and arose through duplications of a single ancestral opsin gene. Mutations in duplicated genes can lead to the evolution of additional spectral classes of cone visual pigments within a class of opsin gene. In fact, in many teleost fishes, cone opsin genes have undergone gene duplication to produce a wide range of opsin genes [6-8]. The expression of different subsets of these genes can potentially generate large-scale variation in spectral sensitivity and the mechanisms of color vision in fish [9-11].

The cichlid fishes of Lake Malawi are an excellent model system for examining large-scale variation in color vision. Lake Malawi has 700-800 species of cichlids [12-14] that have evolved from a common ancestor in a brief period of evolutionary time (2-4 million years) [15]. Malawi cichlids are notable for their diversity in male nuptial color patterns, sexual dimorphism in color patterns and visual communication processes governing mate choice [16-18]. Cichlids have undergone multiple opsin gene duplication events, producing seven classes of cone opsin genes [10,19]. These seven opsin genes include an ultraviolet-sensitive (*SWS1*), two short-wavelength-sensitive (*SWS2a *and *SWS2b*), three mid-wavelength-sensitive (*RH2aα*, *RH2aβ*, *RH2b*) and long-wavelength-sensitive (*LWS*) cone opsins. Of particular interest is that Malawi cichlids show differential expression of primarily three of the seven available cone opsin genes [9,20,21], with some evidence of differential expression through ontogeny [20]. This raises an intriguing question of how seven opsin genes are maintained in Malawi cichlids. Our central research focus concerns the large-scale diversity in cichlid visual systems and differences in the number of cone classes that participate in color vision. Here we examine variation in cone classes contributing to spectral sensitivity in cichlids between individuals, sexes and species and discuss the adaptive significance of this variation in color vision.

## Results

To quantify the number of physiologically functional cone classes, we recorded electroretinograms (ERG) from whole fish preparations of three Malawi cichlids: *Metriaclima zebra*, *Melanochromis auratus *and *Protomelas taeniolatus*. We measured the *b-wave *amplitude of ERGs, representative of ON (response to light stimulus onset) bipolar cell activity, to assess the integrative response of functional cones. We were primarily interested in evaluating the number of cone classes contributing to spectral sensitivity and their corresponding visual pigments, but we also used multiple-mechanism modeling to understand the opponent and nonopponent cone interactions at play that can shape the spectral tuning of cone mechanisms. Our analysis was focused on describing variation in spectral sensitivity with respect to individual, sex and species differences.

### Individual differences in spectral sensitivity

**Figure 1:**
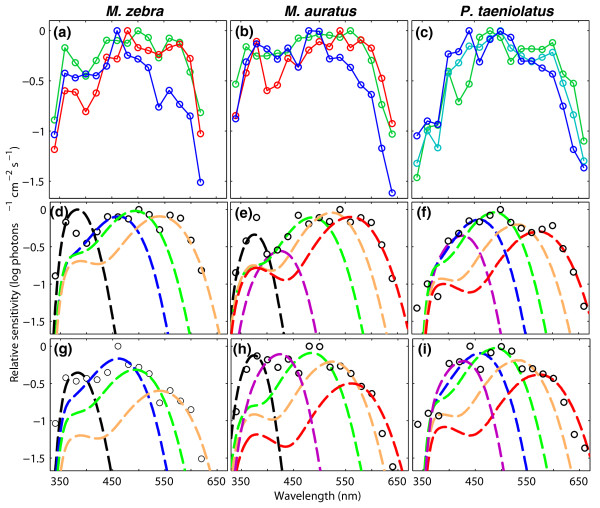
**Relative spectral sensitivity of Lake Malawi cichlids obtained under different background conditions**. **(a-c) **Relative spectral sensitivity of three individuals of *M. zebra*, *M. auratus *and *P. taeniolatus *(left to right). The spectral sensitivity of each species was measured under three background conditions: long-wavelength isolation (LW, red), control (green), and intense short-wavelength isolation (SW, blue). In *P. taeniolatus*, LW was replaced by Dim short-wavelength isolation (Dim-SW, cyan). In *M. zebra*, the spectral sensitivity curves measured under all conditions show four sensitivity peaks, whereas in *M. auratus *and *P. taeniolatus *the complete set of five sensitivity peaks was revealed across all background conditions. Data points were connected with lines to facilitate the identification of sensitivity peaks. **(d-i) **Visual pigment templates (dashed lines) fitted to spectral sensitivity (circles) measured under two background conditions. (Typically, the examination of two spectral sensitivity curves was sufficient to correlate all sensitivity peaks to cone pigments.) *M. zebra*: control (D) and SW (G); *M. auratus*: LW (E) and SW (H); *P. taeniolatus*: Dim-SW (F) and SW (I). Visual pigment templates: SWS1 (black), SWS2b (violet), SWS2a (blue), RH2b (green), RH2a (orange) and LWS (red). Similar plots were used for all individuals to identify sensitivity peaks and correlate them to cone pigments. See criteria for peak identification in the *Methods *section. See Additional file 1 for background isolation conditions and quantum catch of cone pigments.

Over 100 spectral sensitivity curves were recorded from a total of 15 females and 19 males of the three species studied. The spectral sensitivity of each individual was evaluated under three different color background conditions, each aimed at isolating the sensitivity of specific cones (Figure 1; see Additional file 1 for the background conditions and the quantum catches of the various cone mechanisms). For each individual, we identified the number of sensitivity peaks and their spectral locations for the three background conditions. We then fitted cone pigment absorption templates [22] to the sensitivity peaks using a least-squares method.

Individuals of all three species of Malawi cichlids examined in this study possessed 11 different cone subsets (Table 1; see Table 2 for specific λ_max _values and goodness of fit of visual pigment templates). A maximum of five different cone classes were present in 41% of fish, four different cone classes were present in 56% of fish, and three different cone classes were present in only 3% of the fish (one individual) (Table 1). All cone subsets (excluding no. 5) consisted of at least two pigments corresponding to single cones and two pigments corresponding to double cones.

**Table 1:** Cone pigment subsets exhibited by the cichlid species examined

	Subset	Individuals	Visual pigment						Cones	Frequency (%)
					
			SWS1	SWS2b	SWS2a	RH2b	RH2a	LWS		
*M. zebra*	1	3	382 ± 1	427		492 ± 2	537 ± 3		4	27.3
	2	8	380 ± 4		460 ± 2	490 ± 4	535 ± 7		4	72.7

*M. auratus*	3	4	374	424		485	525	561	5	36.4
	4	1	380		463	494	543		4	9.1
	5	1		429		497	547		3	9.1
	6	1	374		457	485	525	561	5	9.1
	7	1		424	457	485	526	562	5	9.1
	8	2		424		485	525	561	4	18.2
	9	1			457	485	525	561	4	9.1

*P. taeniolatus*	6	3	379 ± 1		457	488 ± 3	530 ± 2	567 ± 8	5	25.0
	7	5		428	457	489 ± 2	532 ± 2	574 ± 3	5	41.7
	9	2		428	457	487 ± 1		567 ± 6	4	16.7
	10	1		428	457	494		571	4	8.3
	11	1		428	457		529	571	4	8.3

In total, 11 different subsets of cone pigments were identified across the species studied. The number of individuals that exhibited each pigment subset, the number of cone classes included in each subset and the frequency of each cone pigment subset per species are indicated. The λ_max _(nm) values of cone pigments that comprised each subset are given (means ± SD).

**Table 2:** Statistics of visual pigment templates fit to spectral sensitivity curves

	Gender	Fish ID	Visual pigment template^a^						A_2_%^b^	1^st^ R^2c^	2^nd^ R^2^^d^	Subset^e^
							
			SWS1	SWS2b	SWS2a	RH2b	RH2a	LWS				
*M. zebra*	Female	ZF7	381 (0.62)	427 (0.63)		491 (0.76)	535 (0.90)		37.6	RH2a (0.98)	LWS (0.47)	1
		ZF6	381 (0.46)	427 (0.82)		491 (0.70)	535 (0.90)		37.6	RH2a (0.98)	LWS (0.21)	1
		ZF8	383 (0.88)	428 (0.55)		494 (0.73)	540 (0.87)		53.8	RH2a (0.94)	LWS (0.59)	1
		ZF1	383 (0.09)		461 (0.96)	493 (0.72)	540 (0.85)		48.4	RH2a (0.95)	LWS (0.58)	2
		ZF4	379 (0.91)		459 (0.30)	487 (0.69)	530 (0.94)		21.5	RH2a (0.93)	LWS (0.01)	2
		ZF2	375 (0.53)		461 (0.98)	493 (0.38)	540 (0.96)		48.4	RH2a (0.95)	LWS (0.56)	2
	
	Male	ZM2	375 (0.85)		457 (0.76)	484 (0.85)	526 (0.91)		8	RH2a (0.89)	LWS (0.49)	2
		ZM6	378 (0.75)		458 (0.57)	487 (0.81)	530 (0.95)		18.8	RH2a (0.97)	LWS (-0.18)	2
		ZM4	383 (0.15)		463 (0.64)	494 (0.54)	540(0.91)		53.7	RH2a (0.99)	LWS (0.62)	2
		ZM1	385 (0.55)		463 (0.99)	495 (0.93)	544 (0.55)		59.1	RH2a (0.91)	LWS (0.64)	2
		ZM3	378 (0.69)		458 (0.18)	487 (0.91)	530 (0.79)		18.8	RH2a (0.91)	LWS (-0.69)	2

*M. auratus*	Female	AF13	374 (0.84)	424 (0.70)		485 (0.82)	525 (0.72)	561 (0.87)	0	LWS (0.85)		3
		AF15	374 (0.25)	424 (0.75)		485 (0.43)	525 (0.14)	561 (0.82)	0	LWS (0.85)		3
		AF14	380 (0.76)		463 (0.33)	494 (0.81)	543 (0.71)		53.7	RH2a (0.91)	LWS (0.24)	4
		AF11		429 (0.47)		497 (0.63)	547 (0.91)		64.5	RH2a (0.94)	LWS (0.84)	5
	
	Male	AM14	374 (0.71)	424 (0.96)		485 (0.41)	525 (0.70)	561 (0.85)	0	LWS (0.96)		3
		AM13	374 (0.67)	424 (0.60)		485 (0.73)	525 (0.74)	561 (0.85)	0	LWS (0.85)		3
		AM12	374 (0.54)		457 (0.85)	485 (0.41)	525 (0.27)	561 (0.98)	0	LWS (0.98)		6
		AM11		424 (0.32)	457 (0.09)	485 (0.85)	526 (0.43)	562 (0.57)	3	LWS (0.89)		7
		AM16		424 (0.56)		485 (0.41)	525 (0.47)	561 (0.70)	0	LWS (0.84)		8
		AM17		424 (0.97)		485 (0.70)	525 (0.29)	561 (0.88)	0	LWS (0.96)		8
		AM18			457 (0.52)	485 (0.33)	525 (0.46)	561 (0.87)	0	LWS (0.87)		9

*P. taeniolatus*	Female	TF6		428 (0.49)	457 (0.89)	491 (0.75)	533 (0.55)	576 (0.95)	18.1	LWS (0.86)		7
		TF2		428 (0.66)	457 (0.98)	487 (0.84)	528 (0.55)	571 (0.43)	10.6	LWS (0.63)		7
		TF1		428 (0.49)	457 (0.97)	48 (0.49)		571 (0.91)	15.1	LWS (0.98)	RH2a (0.82)	10
		TF4		428 (0.48)	457 (0.54)		529 (0.88)	571 (0.72)	10.6	LWS (0.91)		11
		TF7	379 (0.81)		457 (0.47)	487 (0.63)	529 (0.54)	563 (0.73)	6	LWS (0.93)		6
	
	Male	TM1		428 (0.68)	457 (0.64)	491 (0.59)	533 (0.52)	576 (0.88)	18.6	LWS (0.96)		7
		TM3		428 (0.71)	457 (0.08)	494 (0.89)	529 (0.59)	571 (0.98)	15	LWS (0.95)		7
		TM8		428 (0.59)	457 (0.94)	491 (0.90)	534 (0.49)	576 (0.80)	22.6	LWS (0.95)		7
		TM4		428 (0.64)	457 (0.91)	486 (0.84)		563 (0.98)	3	LWS (0.98)	RH2a (0.96)	9
		TM6		428 (0.42)	457 (0.45)	487 (0.74)		571 (0.94)	10.7	LWS (0.97)	RH2a (0.87)	9
		TM5	379 (0.78)		457 (0.84)	486 (0.50)	529 (0.57)	563 (0.91)	3	LWS (0.97)		6
		TM2	380 (0.95)		457 (0.99)	491 (0.67)	533 (0.87)	576 (0.99)	18.1	LWS (0.99)		6

^a^λmax (nm) of Visual pigment template that were fitted to the sensitivity curve (*R*^2 ^values of the template fits in parentheses). Owing to cone interactions, the sensitivity peaks measured were typically narrower than the fitted pigment templates, resulting in relatively low *R*^2 ^values.

^b^Estimated A_2 _percent in the retina (A_2 _percent = A_2 _proportion × 100).

^c^The visual pigment template that resulted in the highest *R*^2 ^value when fitted to the long-wavelength limb of the spectral sensitivity curve (*R*^2 ^value of the template fit in parentheses).

^d^The visual pigment template which resulted in the second highest *R*^2 ^value when fitted to the long-wavelength limb of the spectral sensitivity curve (*R*^2 ^value of the template fit in parentheses).

^e^The cone pigment subset to which each individual was assigned.

Examining the individuals of each species separately revealed that all individuals in *M. zebra *possessed four cone pigments, while 55% of individuals in *M. auratus *and 67% of individuals in *P. taeniolatus *possessed five cone pigments (Table 1).

The collection of all cone subsets exhibited by either *M. auratus *or *P. taeniolatus *included the complete opsin gene set (Table 1). That is, each of these species utilizes the complete set of six cone pigments (RH2aα and RH2aβ pooled because of genetic and functional similarity [10,19]). However, the collection of all subsets exhibited by *M. zebra *encompassed only five cone pigments and excluded the LWS pigment.

### Sex differences in spectral sensitivity

To evaluate how sex differences contribute to the variation in cone subsets, we calculated the frequency of cone subsets in each species and sex. In all species, the individual variation in pigment subsets used was high, and a strong trend for differences between females and males in the identity and frequency of the pigment subsets they utilized emerged (Table 3). The degree to which pigment subsets were shared between sexes varied across species. Half of *M. zebra *females shared subset 2 with all conspecific males, 28% of *M. auratus *males shared subset 3 with half the females and 60% of *P. taeniolatus *females shared subsets 6 and 7 with 71% of males.

**Table 3:** Frequency of cone pigment subsets across species and sex

		Frequency (%)					
		
		*M. zebra*		*M. auratus*		*P. taeniolatus*	
**Subset**	**Cones**	**Female (6)**	**Male (5)**	**Female (4)**	**Male (7)**	**Female (5)**	**Male (7)**

1	4	50.0	-	-	-	-	-
2	4	50.0	100.0	-	-	-	-
3	5	-	-	50.0	28.6	-	-
4	4	-	-	25.0	-	-	-
5	3	-	-	25.0	-	-	-
6	5	-	-	-	14.3	20.0	28.6
7	5	-	-	-	14.3	40.0	42.9
8	4	-	-	-	28.6	-	-
9	4	-	-	-	14.3	-	28.6
10	4	-	-	-	-	20.0	-
11	4	-	-	-	-	20.0	-

Frequency of cone pigment subsets in females and males of *M. zebra*, *M. auratus *and *P. taeniolatus*. Frequency of cone pigment subsets was calculated for each sex and each species on the basis of spectral sensitivity data. The sample size for each of the examined groups is indicated in parentheses.

**Figure 2:**
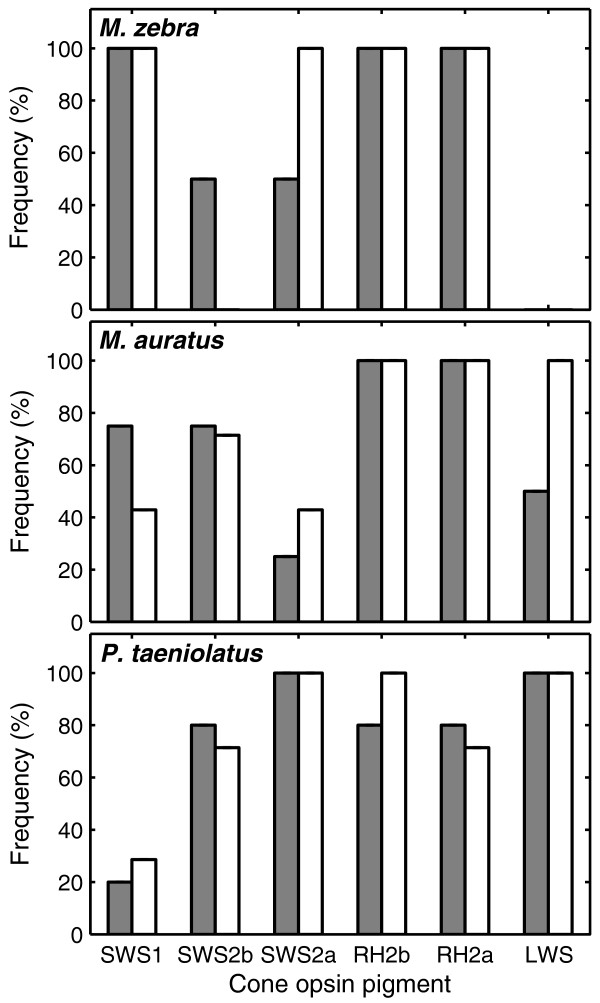
**Frequency of cone pigments across sex**. Frequency of cone pigments in females (gray) and males (white); *M. zebra *(top), *M. auratus *(middle) and *P. taeniolatus *(bottom). Frequency of cone pigments was calculated for each sex and each species based on spectral sensitivity data. For example, three of four *M. auratus *females possessed the SWS1 cone pigment in their retinas, thus 75% of *M. auratus *females possessed the SWS1 cone pigment. On the other hand, three of seven *M. auratus *males possessed the SWS1 cone pigment in their retina, thus 43% of *M. auratus *males possessed the SWS1 cone pigment. Sex differences in the frequency of cone pigments in *M. zebra *and *M. auratus *are larger than in *P. taeniolatus*.

The frequency of each cone pigment present in females and males was calculated (Figure 2). Sex differences in the frequency of cone pigments were evident in all species, with *M. auratus *and *M. zebra *showing the largest differences. The frequency of *M. auratus *females showing SWS1 pigments was 75% larger than in males, while the frequency of males showing SWS2a and LWS pigments was 71% and 100% larger than in females, respectively. The frequency of *M. zebra *males showing SWS2a cone pigment was 100% larger than in females, while SWS2b cone pigment was found only in females. In contrast, the frequency of cone pigments in *P. taeniolatus *showed less variation across sexes, with pigment frequencies differing 12%-42% between sexes.

**Figure 3:**
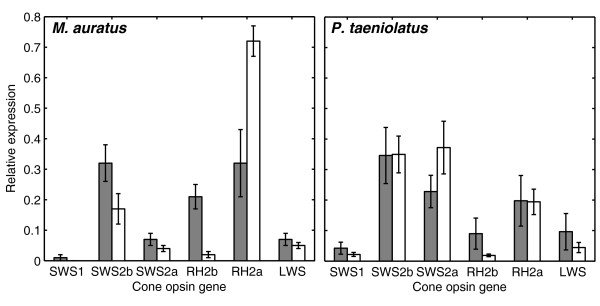
**Relative cone opsin gene expression across sex**. Relative expression of cone opsin genes (means ± SEM) in females (gray) and males (white), *M. auratus *(left) and *P. taeniolatus *(right). Female and male *M. auratus*, but not *P. taeniolatus*, differed significantly in the expression of the *RH2b *and *RH2a *opsin genes. See Additional file 2 for detailed *t*-test results and Additional file 3 for primer specifications for cone opsin genes.

We also examined the effect of sex on the pattern of cone opsin gene expression. We focused our analysis on *M. auratus *and *P. taeniolatus*, the species that exhibited the lowest and highest degree of common cone pigment subsets between the sexes, respectively. Variation in opsin gene expression between females and males in *M. auratus *was larger than in *P. taeniolatus *(Figure 3). Specifically, in *M. auratus*, the expression of *RH2b *and *RH2a *opsin genes differed across sexes (*t*-test, *df *= 8, *P *< 0.0002 and *P *< 0.005, respectively). In contrast, in *P. taeniolatus*, no significant differences in the relative opsin gene expression were detected between sexes (*t*-test, *P *> 0.2 for all genes). See Additional file 2 for detailed *t*-test results and Additional file 3 for primer specifications for cone opsin genes. The frequency of cone pigments and opsin gene expression are not quantitatively comparable. In the calculation of pigment frequency, each individual is scored as either 1 (pigment present) or 0 (pigment absent), whereas gene expression can assume any value between 0 and 1. Moreover, the expression level of an opsin gene does not necessarily dictate its contribution to sensitivity, since patterns of convergence of cones onto retinal interneurons and the network processing of these neurons [23] are critical to governing visual responsiveness. Our results show that females and males differ in the cone opsin gene expression profiles. Our results also suggest that females and males differ in cone pigment subsets and in the frequency of cone pigments. However, owing to sample size limitation imposed by the complexity of spectral sensitivity measurements, the statistical significance of the sex differences observed in cone pigment subsets and in the frequency of cone pigments cannot be evaluated at this time. It is important to note, however, that the variability of all three properties of the visual system of cichlids was consistently larger in *M. auratus *than in the other two species.

### Species differences in spectral sensitivity

In general, different cichlid species used different cone subsets (Table 3). Closer examination reveals that females of the three species did not use the same cone subsets, but 43% of male *M. auratus *shared three cone subsets with *P. taeniolatus *males.

**Figure 4:**
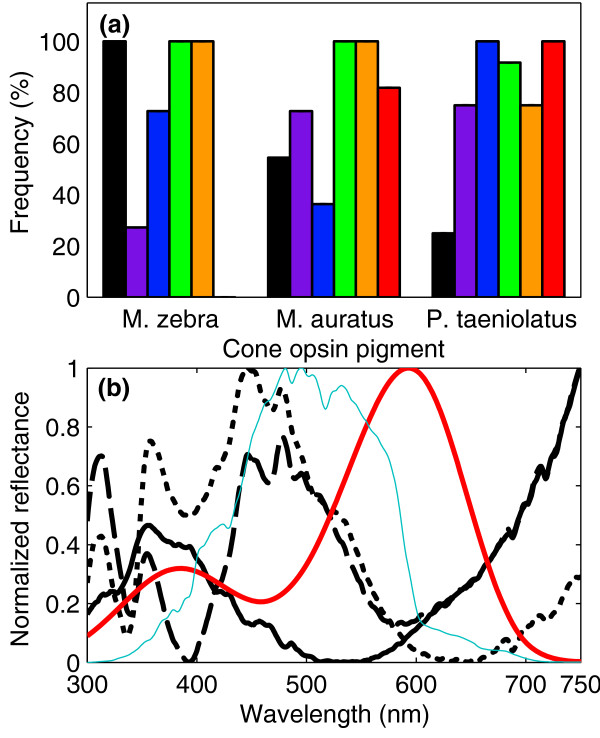
**Frequency of cone pigments and spectral reflectance of the color pattern of cichlids**. **(a) **Frequency of cone pigments in *M. zebra*, *M. auratus *and *P. taeniolatus*. Visual pigments: SWS1 (black), SWS2b (violet), SWS2a (blue), RH2b (green), RH2a (orange) and LWS (red). Cone pigment frequency varied across species, with LWS and SWS1 showing the largest frequency variation between species. The frequency of SWS1, SWS2a and LWS differed significantly across species (Fisher's exact test, *P *= 0.0005, *P *= 0.002 and *P *= 0.00001, respectively). The frequency of SWS2b, RH2a and RH2b did not differ significantly across species (Fisher's exact test, *P *= 0.05, *P *= 0.09 and *P *= 1, respectively). Error rate was set to α = 0.0083 following Bonferroni correction for six hypothesis tests. **(b) **Average normalized spectral reflectance (n = 10; black lines) of the color pattern of *M. zebra *(solid line), *M. auratus *(dashed line) and *P. taeniolatus *(dotted line). The normalized absorption of LWS visual pigment (red line) and the normalized sidewelling irradiance measured at 5-m depth in Lake Malawi (blue thin line) are also depicted.

The frequency of cone pigments varied across species (Figure 4a). The frequency of RH2a (SD = 14%) and RH2b (SD = 5%) showed the smallest variation and did not differ significantly between species (Fisher's exact test; *P *= 0.091 and *P *= 1, respectively). In contrast, the frequency of LWS (SD = 53%) and SWS1 (SD = 38%) showed the largest variation and differed significantly between species (Fisher's exact test; *P *< 0.001 for both pigments). See Figure 4 caption for detailed statistics. The frequency of SWS1 was highest in *M. zebra *(100%), lower in *M. auratus *(54%) and lowest in *P. taeniolatus *(25%). The frequency of LWS in *P. taeniolatus *(100%) and *M. auratus *(81%) was higher than in *M. zebra *(0%).

The frequency of the SWS1 cone pigment across species qualitatively correlated with planktivory. *M. zebra *may be highly planktivorous, feeding on both phytoplankton (mainly diatoms) and zooplankton (Cladocerans and copepods) [24]. *M. auratus is *omnivorous, consuming algae and a wide range of nonalgal dietary items such as cyclopoid copepods [24,25]. *P. taeniolatus *is almost strictly herbivorous and feeds on the biocover detached from rocks, mainly comprising algae and diatoms [21,26].

The frequency of the LWS cone pigment across species was quantitatively correlated with the proportion of long-wavelength reflectance in the color pattern of conspecific males (Figure 4b). As a first-order approximation, the quantum catch of an LWS cone pigment was calculated for the spectral reflectance of the species used in this study. The normalized quantum catch was highest in *P. taeniolatus *(100%), lower in *M. auratus *(80%) and lowest in *M. zebra *(17%).

### Color vision in Lake Malawi cichlids

Spectral sensitivity curves determined using different color background conditions were used to evaluate mechanisms of color vision. Color vision requires the possession of at least two differentially sensitive cones that interact through opponent and nonopponent processes to enable wavelength discrimination [27]. Our results show that 97% of individuals possessed four or five cone classes. To study cone interactions, we used a multiple-cone mechanism (MCM) model, employing strict criteria (see Methods), to reconstruct spectral sensitivity under different background conditions. To do this, we selected three individual fish that exhibited the most frequent cone subset for a given species. These were subsets 2, 3 and 7, occurring in 73%, 36% and 42% of the *M. zebra*, *M. auratus *and *P. taeniolatus *individuals, respectively.

**Figure 5:**
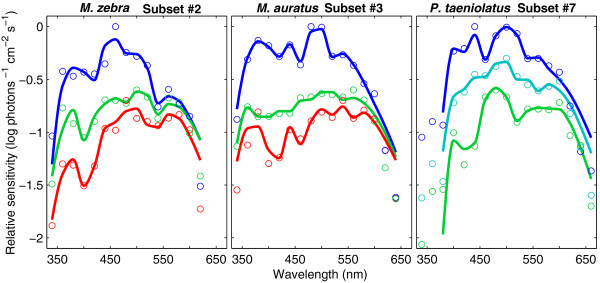
**Spectral sensitivity of cichlids reconstructed using the multiple-cone mechanism model**. Measured (circles) and modeled (lines) relative spectral sensitivity for subsets 2 (*M. zebra*, left), 3 (*M. auratus*, middle) and 7 (*P. taeniolatus*, right). The low sensitivity at 340-380 nm in subset 7 corresponds to β-band absorption of the RH2b, RH2a and LWS pigments and was not modeled. Relative spectral sensitivity of each species was measured under three background conditions: long-wavelength isolation (LW, red), control (green) and short-wavelength isolation (SW, blue). In *P. taeniolatus*, LW was replaced by Dim short-wavelength isolation (Dim-SW, cyan).

Spectral sensitivity was successfully reconstructed using the MCM model (*R*^2 ^= 0.83-0.99 for all species and background conditions) (Figure 5). Cone interaction was expressed as an additive (nonopponent) or subtractive (opponent) contribution to the modeled spectral sensitivity, and we summarize these cone interactions in Table 4 (see Additional file 4 for more comprehensive information that details the specific coefficients and goodness of fit of the model). Typically, the cone class exhibiting the highest sensitivity for a spectral range contributed positively to the modeled spectral sensitivity, whereas other cone classes contributed either positively (nonopponent) or negatively (opponent) to the modeled spectral sensitivity. The successful reconstruction of spectral sensitivity curves further validated our analysis and the distribution of the cone pigment subsets. Most importantly, we identified and characterized cone interactions representative of retinal neural network processing, essential to color vision.

**Table 4:** Summary of nonopponent and opponent cone interactions

		Single cones			Double cones		
	**Spectral range (nm)**	**K_SWS1_**	**K_SWS2b_**	**K_SWS2a_**	**K_RH2b_**	**K_RH2a_**	**K_LWS_**

*M. zebra*	340-400	**+**		-			
(Subset 2)	400-480	-		**+**	-		
	480-540			-	**+**	-	
	540-620				-	**+**	

*M. auratus*	340-380	**+**	-				
(Subset 3)	400-440	-	**+**				
	440-480		-		**+**		
	480-540				-	**+**	
	540-640				**+**		**+**

*P. taeniolatus*	380-420		**+**		-		
(Subset 7)	420-460			**+**	-		
	460-540		-	-	**+**		
	540-580					**+**	**+**
	580-660				-		**+**

K_SWS1_-K_LWS _represent the weights of the relative contribution of each cone mechanism to spectral sensitivity used in the multiple-cone mechanism model. Weights can be positive (nonopponent) or negative (opponent). The spectral ranges listed in this table represent discernible peaks (or cone mechanism) in the spectral sensitivity curves, and each peak was fitted by the multiple cone mechanism model.

## Discussion

### Pentachromatic color vision in Lake Malawi cichlids

Ninety-seven percent of fish examined in this study employed cone subsets of four or five pigments, with most *M. auratus *and *P. taeniolatus *exhibiting five functional cone classes. The spectral sensitivity of all three species of cichlids was shaped by both opponent and nonopponent cone interactions, and thus our data provide the first comprehensive evidence for pentachromatic color vision in vertebrates. Although the presence of five (or more) cone classes was previously reported in microspectrophotometry studies [10,28], this is the first demonstration that five cone mechanisms were used by a single individual. In this regard, designating the dimensionality of color vision requires the essential evidence that shows not only that the different cone mechanisms participate in spectral sensitivity but also that the different cone mechanisms show multiple neuronal interactions. *P. taeniolatus *and *M. auratus *showed five distinct cone classes spectrally compressed in a range of 146 nm (subset 7) and 187 nm (subset 3), respectively. The spectral compression of this sensitivity provides the basis for extraordinary spectral discrimination, which could be important in visual communication. To better understand how pentachromatic color vision would be beneficial to cichlids, we need to consider factors at play in the visual environment of cichlids where visual communication takes place: (1) the high species diversity of cichlids in Lake Malawi (700-800 species) [14,25,29], (2) the large within-genera variation in the color pattern of fish and the small within-genera variation in their size and shape [30-32] and (3) the prevalence of visual cues driving mate choice [33,34] and intrasexual competition [35]. Visual communication would depend on exquisite sensory performance in this visual environment, since cichlids are continually challenged to make fine-scale discriminations of complex color patterns. This visual system capability would be particularly important in guiding visual behavior related to mate choice, where the detection and recognition of conspecific optical signals is critical. These visual adaptations could be fundamental to mate choice fidelity and contribute to the maintenance of biodiversity in the cichlid communities of Lake Malawi.

It is important to point out that our findings differ from the notion that Lake Malawi cichlids primarily exhibit three cone pigments [10,36]. Recent reports show a significant variation in the number of opsin genes expressed among Malawi species [37], with several species (one of which is *P. taeniolatus*) expressing more than three cone opsin genes [21]. Furthermore, microspectrophotometry studies have revealed classes of cones in low abundance in the retina of various species that are of unknown significance [10,36]. Our study suggests that these rare cone classes contribute to the spectral sensitivity of Lake Malawi cichlids.

### Sex differences in the color vision of Lake Malawi cichlids

Our study provides some evidence for sex differences in opsin gene expression, and possibly in spectral sensitivity, for cichlids. While the sample size in electrophysiology studies tends to be low, a possible pattern of differentiation between males and females emerged. The large diversity of cone pigment subsets found in each species was in part qualitatively related to sex differences. This, together with qualitative sex differences in pigment frequency and significant differences in cone opsin gene expression, suggests the possibility for sex differences in the visual system of some Lake Malawi cichlids. Our results suggest that the most prominent sex differences occurred in *M. auratus*. We argue here that males and females may have quite different visual requirements in visual communication scenarios [38-40]. *M. auratus *differs from the two other species in two aspects. (1) *M. zebra *and *P. taeniolatus *males hold a permanent territory while females choose among potential mates [41]; *M. auratus *males, on the other hand, assume a territory only temporarily around breeding events [25]. (2) *M. zebra *and *P. taeniolatus *males are conspicuous while the color pattern of females is dull, whereas both female and male *M. auratus *are conspicuous and exhibit colorful, but very different, body patterns [25].

We propose a possible explanation for the observed distribution of sex differences across species. Considering the significant role of visual cues in female mate choice [33,34] and male-male competition for territory [35], both females and territorial males of *M. zebra *and *P. taeniolatus *would likely benefit from possessing color vision attuned to provide the best recognition ability of conspecific males. In contrast, *M. auratus *males assume a territory only temporarily and thus are less dependent on recognition of conspecific males to increase their mating success. Additionally, not holding a permanent territory potentially allows *M. auratus *males to choose between females. Indeed, in the cichlid *Astatotilapia flaviijosephi*, males defend territories only during the breeding season and actively choose between females [42]. Furthermore, the investment of *M. auratus *females in their color pattern adds support to the hypothesis that *M. auratus *males choose between females. Thus, the distribution of sex differences in the visual system across species might be associated with territorial behavior. However, the adaptive role of sexual dimorphism in color vision of Lake Malawi cichlids requires further examination.

### Adaptive significance of species differences in the color vision of Lake Malawi cichlids

The variation in cone pigment frequency across species reflects visual adaptations that both enhance the contrast of transparent prey and detect signals important for mate choice, suggesting that both natural selection and sexual selection played a role in shaping the spectral sensitivity of cichlids.

The frequency of the SWS1 cone pigment across species qualitatively correlated with planktivory. UV photoreception mediated by the SWS1 cone pigment enhances the contrast of transparent zooplankton against the water background and thus aids in their detection [43,44]. However, UV sensitivity does not confer any advantage over longer-wavelength sensitivity in the detection of opaque food items such as loose algae. Thus, UV sensitivity is expected to be highly advantageous for the planktivorous *M. zebra*, less advantageous for the omnivorous *M. auratus *and least advantageous for the herbivorous *P. taeniolatus*, which is in agreement with our findings. Thus, the presence of the SWS1 pigment in the retina of cichlids has likely significant adaptive value. In this regard, the expression of the *SWS1 *opsin gene was recently reported to be highest among species foraging on zooplankton, phytoplankton and algae and lowest among species foraging on fish or benthic invertebrates [21]; however, the relationship between opsin gene expression and spectral sensitivity in cichlids is still largely unknown. UV photoreception is also tightly linked to polarization sensitivity, and this may provide yet greater contrast enhancement of transparent prey [45-47]. Researchers in our lab are currently examining polarization sensitivity in cichlid fishes and its role in visual behavior.

The frequency of the LWS cone pigment across species was quantitatively correlated with the proportion of long-wavelength reflectance in the color pattern of conspecific males. While the possession of the LWS cone pigment by *M. auratus *and *P. taeniolatus *may facilitate the detection of long-wavelength signals of conspecifics, this would not be the case for *M*. zebra that do not have prominent long-wavelength signals, which is in agreement with our findings. Therefore, the presence of the LWS pigment in *M. auratus *and *P. taeniolatus *has a significant adaptive value. Interestingly, the detection of UV reflections from the color pattern of these fish species may be mediated through the β-band of the LWS cone pigment (Figure 4b), thus potentially eliminating the need for the possession of the SWS1 pigment to detect UV signals from conspecifics.

Both natural and sexual selection work sequentially to contribute to the divergence of Lake Malawi rock-dwelling cichlids [16]. In the first episode of cladogenesis, competition for trophic resources resulted in the differentiation of trophic morphology and the diversification of the visual system to allow the utilization of different foraging styles. The correlation of the frequency of the SWS1 cone pigment across species to planktivory (this study), and the recent report that the expression of the *SWS1 *opsin gene correlates with planktivory [21], support the idea of diversification of the visual system based on competition for trophic resources. In the second episode of cladogenesis, sexual selection contributed to the differentiation of male nuptial coloration and the accompanying diversification of the visual system to allow high recognition ability of conspecifics. The correlation of the frequency of the LWS cone pigment across species to the proportion of long-wavelength reflectance from male color patterns supports the idea of visual system diversification based on the diverse male nuptial coloration.

Different cichlid species used different cone subsets, with the spectral sensitivity of females being more constrained than that of males. This is suggestive of specific tuning of color vision in females to allow for the best recognition ability of the distinctly colored conspecific males. Previous studies have shown that different cichlid species express diverse opsin gene subsets [9,21], supporting our results. However, a close examination of the different constraints imposed on the visual systems of females and males needs to be undertaken.

### The retention of cone opsin genes in Lake Malawi cichlids

None of the individuals examined possessed all available six cone pigments (RH2aα and RH2aβ pooled). However, the large number of pigments utilized and the high diversity in pigment subsets within a species add support for the retention of the complete set of cone opsin genes in Malawi cichlids.

Several factors may contribute to the observed pigment diversity within a species. First, a significant genetic component of opsin expression was reported [48] where individuals with different genetic histories may utilize different pigment subsets. Second, thyroid hormones (TH) are important in vertebrate development and can modulate the opsin gene expression and induce cone loss in fish [49,50]. The level of TH in fish has been associated with environmental stress and subordinance via the effect of cortisol [51,52]. Thus, individuals differing in their stress level or social status may utilize diverse pigment subsets through TH level modulation. Third, social visual cues in fish modulate the activity of GnRH neurons [53]. Since GnRH has been shown to affect retinal neurons [53], it is possible that it may be responsible for changes in opsin gene expression and spectral sensitivity in fish.

## Conclusions

The cichlid model system illustrates that the visual systems of fish may differ across individuals, sexes and species. The large number of available cone opsin genes facilitates this variation in the spectral sensitivity of fish. We show that even rare cone populations and opsin gene expression at low levels contribute to the spectral sensitivity of fish. Our results suggest that the diversification of color vision across species contributes to sensory adaptations that both enhance the contrast of transparent prey and the detection of optical signals of conspecifics. Therefore, both natural and sexual selection may work in concert to shape spectral sensitivity in fish. Taken together, our findings have important implications for understanding the variable nature of fish color vision and the selective forces shaping detection and recognition capabilities.

## Methods

### Animals and holding conditions

Three species of Lake Malawi cichlids were used in this study: *Metriaclima zebra *(blue top manda), *Melanochromis auratus *and *Protomelas taeniolatus *(Old World Exotic Fish, Homestead, FL, USA). *M. zebra *and *M. auratus *belong to a rock-dwelling evolutionary lineage, the mbuna, whereas *P. taeniolatus *is a member of the nonmbuna lineage. These species are sexually dimorphic and occur sympatrically in the rocky habitat in Lake Malawi. The males have distinctive nuptial color patterns, and they use the rocky habitat differently with respect to reproductive behavior [25,29]. Adult fish were held in our aquatic facility tanks under a 12 h:12 h light-dark photoperiod at 25 ± 1°C. Facility lighting featured enhanced full spectrum fluorescent lamps (UV-Blue actinic and BlueMax lamps; Full Spectrum Solutions, Jackson, MI, USA). All experimental and animal care procedures were approved by Queen's University Animal Care Committee under the auspices of the Canadian Council for Animal Care.

### Electroretinogram (ERG) experimental apparatus

The general design of the optical system and recording apparatus has been described previously [2,54]. Two background channels each with a 250-W quartz-halogen lamp (Osram, Danvers, MA, USA) were used to provide constant background, to which test fish were light-adapted. Long- and short-pass interference filters (Fused Silica, OD 2; Edmund Optics, Barrington, NJ, USA), band-pass interference filters (Edmund Optics), broadband color filters (Schott, Elmsford, NY, USA) and reflective neutral density filters (Edmund Optics), were used to manipulate the spectral irradiance of each background channel. Light from the two background channels was guided to the electrophysiology rig using a bifurcated optical fiber (fused silica, numerical aperture (NA) = 0.22; Fiberoptic Systems, Simi Valley, CA, USA).

The stimulus channel used a 300-W xenon arc lamp system (Thermo Oriel, Stratford, CT, USA). The optical path consisted of a monochrometer (Instruments SA, Metuchen, New Jersey, USA), Inconel quartz neutral density wedge (0-4.0 neutral density; CVI Melles Griot, Albuquerque, NM, USA), shutter (Uniblitz, Rochester, NY, USA), optical filters to block spectral sidebands and UV lenses to match the numerical aperture of the liquid light pipe (fused silica; NA = 0.55; Fiberoptic Systems). The background and stimulus optical fibers were fitted to a beam splitter to produce a stimulus beam (diameter 0.5 cm at the plane of the fish eye) contained within the background beam (diameter 1 cm). This setup ensured that the chromatically adapted portion of the fish retina was also the one stimulated.

### Preparation of fish

Prior to ERG recordings, fish were immersed in a solution of 150 mg l^-1 ^tricaine methanesulfonate (MS-222) until the fish reached stage IV anesthesia [55]. Intramuscular injections of a general anaesthetic, Maranil (0.1 mg g^-1 ^body mass) and an immobilizing agent, pancuronium bromide (0.04 mg g^-1 ^body mass), were administered at several sites. Test fish were then placed in a holding cradle in a Faraday cage. Experimental fish were irrigated with aerated fresh water (20°C, flow rate ~3 ml s^-1^), and their body was covered with a moist cloth.

### ERG recording procedure

ERG recordings started at least 1 hour following the onset of the light phase and concluded before the onset of the dark phase to avoid any effects related to circadian rhythm [2,56,57]. A glass electrode (1.5 mm outer diameter, 1 mm inner diameter, borosilicate glass; World Precision Instruments, Sarasota, FL, USA) that was pulled to a tip diameter of 80-125 μm (P-97 Flaming/Brown Micropipette puller; Sutter Instruments, Novato, CA, USA) was loaded with saline (0.684 M sodium chloride) and inserted into a saline-filled chlorided AgCl half-cell (A-M systems, Sequim, WA, USA). The electrode tip was positioned using a micromanipulator on the dorsal-nasal surface of the right eye. A ground electrode was attached to the caudal fin and a chlorided-silver reference electrode was placed on the head of the test fish. Fish were chromatically adapted for 1 hour prior to experiments. The stimulus duration was 500 ms with an interstimulus interval of 5 s. An isolated bioamplifier (ISO-80; World Precision Instruments, Sarasota, FL, USA) amplified the ERG signal and filtered the signal using band-pass filter settings (5-Hz low-pass, 100-Hz high-pass). The amplified signal was analyzed with a 16-bit A/D data acquisition system and Signal 4.0 software (Micro 1401; Cambridge Electronic Design Limited, Cambridge, UK). A custom-designed software analysis module determined the b-wave amplitude (corresponding to the ON response of bipolar cells) by measuring the potential change between the a-wave and b-wave peaks. Spectral sensitivity was measured in 20-nm increments, from 320 to 700 nm, using a staggered wavelength presentation to prevent adaptation to a spectral region.

### Analysis of electroretinograms

A response versus intensity (RI) curve was generated for each wavelength examined. To determine the sensitivity at a given wavelength, the empirical Naka-Rushton function was least-squares fitted to the RI curve with a slope parameter of 1 [58,59]. The log irradiance level corresponding to half response (LogK) was determined, from which the sensitivity was calculated by taking the reciprocal of this value. Typically, the upper asymptote of the Naka-Rushton function could be reached for all wavelengths. There were a few exceptions at wavelengths shorter than 360 nm and longer than 600 nm. At these wavelengths, the value of the maximum response parameter (R_max_) was set to that of the neighboring wavelength examined, prior to fitting. A log relative sensitivity curve was constructed by normalizing the log absolute sensitivity values to the maximum sensitivity. This procedure was repeated for each individual under each of the background conditions.

### Background isolation conditions

Four background conditions were designed and used for isolating cones dominating certain spectral regions. A long-wavelength-isolation condition (LW) was used for isolating cone mechanisms most sensitive to long wavelengths, dim and intense short-wavelength-isolation conditions (Dim-SW and SW) aimed at isolating cone mechanisms most sensitive to short wavelengths and a relatively spectrally flat background condition was used as a control. To design and carefully control the level of light adaptation of the respective cone mechanisms, a quantum catch model was used:

(1) $$Q_{i}=\int_{300}^{800}A_{i}(\lambda )E_{i}(\lambda )d(\lambda )$$

where *Q*_*i *_(photons cm^-2 ^s^-1^) denotes the quantum catch of cone mechanism *i *(*i *= 1, 2, ..., 6), *A*_*i*_(λ) represents the visual pigment absorption coefficient of cone mechanism *i *at a wavelength λ (nm), and *E*_*i*_(λ) denotes the photon irradiance of the background light field at a wavelength λ.

### Characterization of background conditions

The irradiance provided under the various background conditions was characterized by measuring the spectral irradiance of the background beam. Spectral irradiance was measured using a spectroradiometer (QE65000; Ocean Optics, Dunedin, FL, USA) connected to a 2-m optical fiber (QP600-2-UV/VIS; Ocean Optics) that was fitted with a cosine corrector (CC-3-UV; Ocean Optics). The spectroradiometer utilized a 1024 × 58-element square silicon CCD (charge-coupled device) array and was configured with a 25-μm slit and a variable blaze wavelength grating (HC-1, groove density = 300 mm^-1^; Ocean Optics), resulting in an effective spectral resolution of 1.9 nm (FWHM) between 200 and 950 nm. The fiber end was held approximately 5 cm away from the emergence plane of the background beam, ensuring sampling of the entire beam diameter. The spectroradiometer setup was calibrated for absolute irradiance using a NIST (National Institute of Standards and Technology, Gaithersberg, MD, USA) calibrated Halogen-Deuterium dual light source (200-1000 nm, DH-2000-CAL; Ocean Optics). In cases where spectral variation exceeded the dynamic range of the spectroradiometer and thus a reliable measurement could not be obtained, the irradiance delivered under the background condition was calculated by multiplying the measured output of each light source by the spectral transmission of the filters used in producing the background condition.

### Analysis of spectral sensitivity curves

#### 1.	Identification of functional cones

Two criteria were devised for the identification of cone mechanisms. A sensitivity peak was considered to be a cone mechanism if it satisfied the following conditions: (1) the sensitivity peak appeared under at least two background isolation conditions, and (2) the sensitivity peak exhibited changes that were directly related to changes in the spectral composition of the background conditions. For instance, a sensitivity peak in the ultraviolet range (UV; 340-400 nm) was identified as a UV cone when sensitivity increased under a short-wavelength isolation background or as the β-band of mid- and long-wavelength cones when sensitivity decreased under a short-wavelength isolation background.

#### 2.	Correspondence between visual pigments and cone mechanisms

To relate visual pigments with cone mechanisms, absorption templates of visual pigments were fitted to spectral sensitivity curves using a least-squares fit. This technique was previously used in numerous studies and repeatedly has been shown to allow for the identification of the cone classes in the retinas of animals [60-62]. In this regard, interactions between different cone classes produce sensitivity peaks that are narrower than the absorption templates of visual pigments, resulting in a somewhat reduced goodness of fit [60,61]. Visual pigment absorbance templates [22] were constructed for the opsin genes previously reported in cichlids: Note that the λ_max _of A_1_-reconstituted visual pigment is provided in parentheses SWS1 (368 nm), SWS2b (423 nm), SWS2a (456 nm), Rh2b (484 nm), Rh2aα (519 nm), Rh2aβ (528 nm) and LWS (560 nm) [10,19]. In the case of Rh2aα and Rh2aβ, the spectral overlap of the two visual pigment curves necessitated calculating an average λ_max _of 523 nm. The 532-nm λ_max _absorption spectrum was used for subsequent analysis and is hereafter referred to as RH2a. To generate visual pigment templates we combined absorption spectra for the A_1 _and A_2 _chromophores (A_1_, equations 1, 2, 4, 5a, 5b; A_2_, equations 1, 4, 6a, 6b, 8a, 8b) [22]. The proportion of the A_2 _state was presented using a fraction parameter, *a *(0 ≤ *a *≤ 1), and therefore the absorption spectra of a given cone type exhibiting an A_2 _proportion of *a *was calculated as A_2 _(*a*) = A_1_·(1 - *a*) + A_2_·*a*. The λ_max _of each cone type exhibits a defined wavelength shift as the A_2 _proportion changes [63]. This shift was also taken into account when generating the visual pigment templates for varying A_2 _proportions.

Typically, the long-wavelength peak in spectral sensitivity was located between 560 and 600 nm, and thus it could correspond to either the RH2a (523-560 nm; λ_max _A_1_-λ_max _A_2_) or the LWS (560-626 nm; λ_max _A_1_-λ_max _A_2_) visual pigments. Therefore, to correlate the long-wavelength peak with a visual pigment absorption curve, it was necessary to determine the A_2 _proportion in the retina of each individual. Visual pigment absorption templates for the RH2a and LWS pigments were fitted to the long-wavelength limb of the spectral sensitivity curve. The least-squares fit was performed while leaving the A_2 _proportion and a magnitude coefficient unrestricted, allowing the software to find a visual pigment template with an A_2 _proportion that best describes the long-wavelength limb. Estimating the A_2 _proportion on the basis of the long-wavelength limb is the best possible approach because (1) the λ_max _shift between the A_1 _and A_2 _states is largest for long-wavelength-sensitive cones, and (2) the spectral sensitivity is based on one cone class and thus is not affected by opponent interactions. For each individual, the A_2 _proportion estimate was used for fitting all visual pigment templates to the spectral sensitivity curve. All absorption templates were corrected for the spectral transmission of the fish lens (*P. taeniolatus *[64]; *M. zebra *and *M. auratus *measured [1]).

#### 3.	Modeling cone interactions

A multiple-cone mechanism (MCM) model was used to determine the relative contribution of each cone class to each spectral sensitivity curve by assigning weights, which can be positive (excitatory) or negative (inhibitory) for each cone mechanism. This "upper envelope" model that was previously used in primates [65,66] and fish [61] assumes that the spectral sensitivity of the eye is determined by the cones that are most sensitive over the spectral region of concern. This linear model takes the general form S_λ1, λ2 _(λ) = ∑ *k*_*i*_·*A*_*i *_(λ), where *S*_*λ1, λ2*_*(λ) *denotes the modeled spectral sensitivity in the spectral range enclosed by *λ1 *and *λ2 *while accounting for cone interactions; *A*_*i*_*(λ) *represents the absorption of the visual pigment template, corresponding to cone *i *(*i *= 1, 2, ..., 6) at a wavelength *λ *(nm); and *k*_*i *_denotes the weight representing the contribution of cone *i*. The MCM model was least-squares fitted to the spectral sensitivity curves determined under the various background conditions for each individual separately. The spectral range between two sensitivity notches was set as the *λ1 *- *λ2 *range [61,65,66]. For modeling spectral sensitivity, we selected the cone interaction that (1) included the minimum possible number of cones, (2) allowed for the best fit under all background conditions, and (3) ensured the same type of cone interaction (opponent and nonopponent) under all background conditions. To prevent overparameterization of the model, the number of possible cone interactions was restricted to three. That is, the maximum number of free parameters in the model was three, while the number of data points used to fit the model was always larger than three. The weights assigned to each cone, in addition to the *R*^2 ^values (total amount of variance accounted for by the model across the spectrum), were determined. Log relative sensitivity was transformed to percent relative sensitivity and normalized to range between 0 and 1 prior to fitting [67].

### Preparation of retinal samples

Upon completion of ERG recordings, fish were dark-adapted for 1 hour and then killed by cervical transection. Under deep red illumination (>650 nm), both eyes were enucleated and hemisected along an anterior-posterior axis. The neural retina was then dissected free of pigmented epithelium. Each isolated retina was preserved in 0.5 ml of RNAlater (Ambion), stored at 4°C for the first 24 hours, and then kept at -80°C until further processing. The sex of the fish was determined using the acetocarmine technique [68,69]. Fish were dissected and their gonads were removed, stained with acetocarmine and viewed under a dissecting microscope to determine the fish sex. All fish were sexually mature adults showing well-developed eggs or sperm and were at least 9 months old. Cichlids typically reach sexual maturity at 6 months from release [70,71].

### Relative gene expression by qPCR

Quantitative real-time RT-PCR (qPCR) was used to quantify the relative levels of mRNA expression corresponding to the various cone opsin genes. Unless specified otherwise, all procedures described below were performed following the manufacturer's protocols. Total RNA was extracted from retinas (Absolutely RNA Miniprep Kit; Agilent Technologies, Santa Clara, CA, USA), and its amount and quality were determined by reading the absorbance at 260 nm and calculating the absolute 260/280 absorbance ratio using a benchtop spectrophotometer (Cary 300 Bio; Varian, Santa Clara, CA, USA). Total cDNA was synthesized using 250 ng of total retinal RNA and a Superscript III first-strand synthesis SuperMix (Invitrogen, Carlsbad, CA, USA) at 50°C for 30 minutes. See Additional file 3 for primer sequences for the amplification of the cDNA opsin genes in cichlids (*RH2aα *and *RH2aβ *combined). All primers were analyzed using the Primer-Blast primer design tool (National Center for Biotechnology Information, Bethesda, MD, USA) for product size, melting temperature, guanine-cytosine content (%) and sequence specificity. The specificity of all primer pairs was tested by amplifying target sequences present within the cDNA synthesized from retinal total RNA. Amplification was carried out in a Mastercycler gradient 5331 (Eppendorf) using the following concentrations: 2.5 mM MgCl_2_, 200 μM deoxynucleotide triphosphate (dNTP), 100 μM forward and reverse primers, 0.05 U GotTaq Flexi DNA polymerase (Promega, Madison, WI, USA), 1× PCR buffer and 1 μl of cDNA template, in a final volume of 25 μl. The PCR cycling profile consisted of an initial hold at 92°C for 2 minutes, 40 cycles (92°C for 25 s, 55°C for 25 s, 72°C for 25 s), and a final extension at 72°C for 5 min. To verify that the amplified product obtained from each primer pair consisted of a single band and was of the correct size, amplified DNA products were separated in a 2.0% agarose gel 1× TBE (Tris/Borate/EDTA) buffer, visualized by GelRed on a gel imager (AlphaImager, Cell Biosciences, Santa Clara, CA, USA) and compared with a benchtop 100-bp DNA ladder (Promega).

qPCR analysis of individual retinal cDNA samples was carried out for each opsin gene using Brilliant SYBR green qPCR Master Mix (Agilent Technologies) in a real-time quantitative system MX3000P™ (Agilent Technologies). Each 25-μl reaction contained 1× Brilliant SYBR Green Master Mix, 200 mM quantities of both forward and reverse primers, 0.05 μl of ROX passive reference dye and 1 μl of four times diluted cDNA. The reaction mix was placed in 96-well nonskirted polypropylene PCR plate and capped with optical strip caps (Agilent Technologies). The plate was briefly centrifuged and eye-inspected for the presence of bubbles. The thermocycle program was 95°C for 10 minutes, followed by 40 cycles of 95°C for 25 seconds, 55°C for 25 seconds and 72°C for 25 seconds. Controls included a reaction lacking cDNA template) and a nontranscribed reaction for genomic DNA contamination (No-RT). All samples were run in triplicate, and fractional cycle values (Cq) were averaged. Amplification efficiencies per sample per target were calculated from the slope of the amplification curve in the exponential phase using LinRegPCR 11.0 software [72]. Relative gene expression was determined for the six opsin genes as a fraction of the total cone opsin genes expressed for an individual [19]:

(2) $$\frac{T_{g,i}}{T_{all,i}}=\frac{{\left(1+E_{g,i}\right)}^{-Cq_{g,i}}}{\sum {\left(1+E_{g,i}\right)}^{-Cq_{g,i}}}$$

where *T*_*g,i*_*/T*_*all,i *_is the relative gene expression ratio for gene *g *normalized by the total cone opsin genes expressed for individual *i*, *E*_*g,i *_is the amplification efficiency for gene *g *and individual *i*, and *Cq*_*g,i *_is the fractional cycle value for gene *g *and individual *i*. Finally, relative gene expression was averaged for each species and sex.

### Underwater irradiance measurements

Underwater spectral irradiance at a horizontal line of sight was measured in July 2008 at a near-shore site at Cape Maclear on the northwestern part of the Nankumba Peninsula, Lake Malawi (14° 1" 0" S 34° 51" 0" E; local time: 11:00-11:30, GMT +02:00, average solar zenith angle within the water = 25.6°). The sampling site, Otter Point, is exposed to wind and wave action and exhibits a rocky bottom that subsides with depth and becomes a sandy bottom at ~15 m depth [25]. Irradiance was measured using a thermoelectrically cooled spectroradiometer (QE65000; Ocean Optics) connected to a 30-m optical fiber (ZPK600-30-UV/VIS; Ocean Optics) that was fitted with a cosine corrector (CC-3-UV; Ocean Optics). See the specifications of the spectroradiometer in the *Characterization of background conditions *section. Holding the irradiance probe, a SCUBA diver attained position at depths of 5, 10 and 15 m, and readings were saved on a computer placed on a boat (positioned as far as possible and never between the diver and the sun to prevent shading). The spectroradiometer setup was calibrated for absolute irradiance following the same procedure described in the *Characterization of background conditions *section.

### Spectral reflectance measurements

Spectral reflectance of the color pattern of fish was measured using a spectroradiometer (USB2000; Ocean Optics) connected to one arm of a 2-m bifurcated optical fiber (BIF600-2-UV/VIS; Ocean Optics). The other arm of the fiber was connected to a light source (DH-2000-BAL; Ocean Optics). The spectroradiometer utilized a 2048-element linear silicon CCD array and was configured with a 50-μm slit and a grating (groove density, 600 mm^-1^; blaze wavelength, 400 nm; grating 2, Ocean Optics), resulting in an effective spectral resolution of 2.06 nm (FWHM) between 200 and 950 nm. The light source integrated two lamps, tungsten-halogen and deuterium, providing a high and spectrally balanced output between 200 and 1000 nm. The common end of the bifurcated fiber was fitted with a flat black reflectance probe exhibiting a 3-mm diameter tip cut at an angle of 45°. Prior to each measurement session, the light source was allowed to warm up for at least 40 minutes. Then a measurement of a Spectralon diffuse reflectance standard (WS-1-SL; Ocean Optics) was taken as 100% reflectance, and a dark measurement was taken as zero reflectance. Fish were immersed in a 1:10 clove oil:ethanol solution immediately after capture until the fish reached stage IV anesthesia [55]. Fish were held submerged in lake water, and spectral reflectance was measured at 10 points along the trunk of the fish (one individual per species). Care was given to sealing the reflectance probe against the fish skin to reduce stray light. Readings were acquired and saved on a laptop computer.

To calculate the quantum catch of the LWS cone pigment, we used the spectral radiance reflected from the color pattern of the species studied (equation 1). In this case, however, *E*_i_(λ) was substituted by the product of the sidewelling irradiance at a depth of 5 m, *E*_h_(λ) times the spectral reflectance of the color pattern of the fish, *R*(λ). Absorption template for the LWS cone pigment was generated while assuming A_2 _proportion of 0.5 [22].

### Statistical analysis

To study the effect of sex on the relative opsin gene expression, we performed multiple *t*-tests where an experiment-wise error rate of 5% was corrected to 0.83% (α = 0.05/6 = 0.0083) following Bonferroni correction for six hypothesis tests [73] that correspond to six opsin genes (*RH2aα *and *RH2aβ *were pooled because of genetic and functional similarity [10,19]). Prior to performing statistical analyses, the normality of all data was confirmed using the Kolmogorov-Smirnov test, and the homogeneity of variance across treatment groups was confirmed using Cochran's C test [74]. To study the effect of species on the frequency of cone pigments, we performed multiple Fisher's exact tests (two-tailed) with a corrected experimental error (α = 0.0083). Statistical analysis was performed using the Statistica (*t*-test) and R version 2.11.1 (Fisher's exact test) software.

## Abbreviations

CCD: charge-coupled device; Dim-SW: dim short-wavelength-isolation background condition; ERG: electroretinogram; FWHM: full width half max; GnRH: Gonadotropin-releasing hormone; SW: short-wavelength-isolation background condition; LW: long-wavelength-isolation background condition; LWS: long wavelength sensitive; MCM: multiple-cone mechanism; NA: numerical aperture; NIST: National Institute of standards and technology; OD: optical density; RH: rhodopsin-like; RI: response versus intensity; SWS: short wavelength sensitive; TH: thyroid hormones; UV: ultraviolet.

## Competing interests

The authors declare that they have no competing interests.

## Authors' contributions

SS carried out the design of the experiments, executed the spectral sensitivity measurements, performed all data analysis and participated in writing the manuscript; CWH participated in the design of the study and writing the manuscript; RLL performed the qPCR gene expression profiling; SMG performed the spectral reflectance measurements. All authors read and approved the final manuscript.

## Supplementary Material

Additional file 1 **Background isolation conditions for spectral sensitivity measurements**. **(a) **The spectral irradiance provided under the various background isolation conditions. Background conditions: long-wavelength isolation (LW, red), control (green), Dim short wavelength (Dim SW, cyan) and short-wavelength isolation (SW, blue). **(b) **The quantum catches of the six possible cone mechanisms under each condition. Cone pigment: SWS1 (black), SWS2b (violet), SWS2a (blue), RH2b (green), RH2a (orange) and LWS (red). Cone quantum catches were calculated while setting the A_2_% of cones to 50 for the design of the background conditions. Click here for file

Additional file 2 **Summary of *t*-test results for the examination of the effect of sex on opsin gene expression**. Click here for file

Additional file 3 **Primer specifications for cone opsin genes**. Click here for file

Additional file 4 **Statistics of cone interactions for the most frequent cone pigment subset of each species**. Click here for file
