# Supplementary material for: Functional diversity in the color vision of cichlid fishes
Source: BMC Biol. 2010 Oct 28;8:133. doi: 10.1186/1741-7007-8-133 (PMC2988715; doi:10.1186/1741-7007-8-133)

Additional file 1 - Background isolation conditions for spectral sensitivity measurements

(A) The spectral irradiance provided under the various background isolation conditions. Background conditions: Long wavelength isolation (LW, red), Control (green), Dim short wavelength (Dim SW, cyan), and Short wavelength isolation (SW, blue).

(B) The quantum catches of the six possible cone mechanisms under each condition. Cone pigment: SWS1 (black), SWS2b (violet), SWS2a (blue), RH2b (green), RH2a (orange), and LWS (red). Cone quantum catches were calculated while setting the A_2% of cones to 50 for the design of the background conditions.

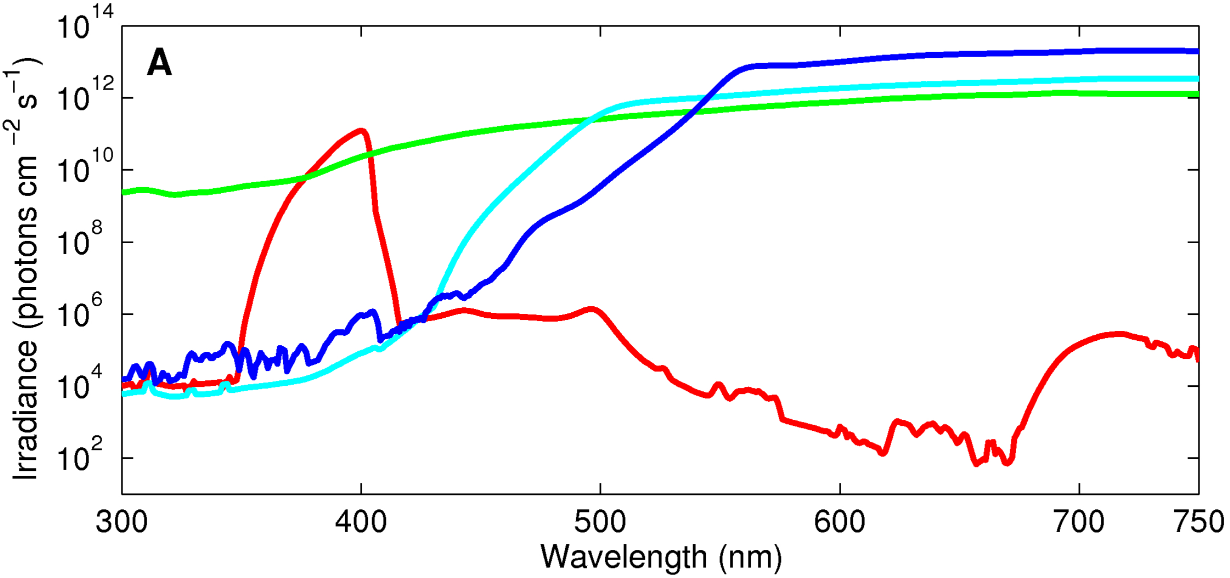

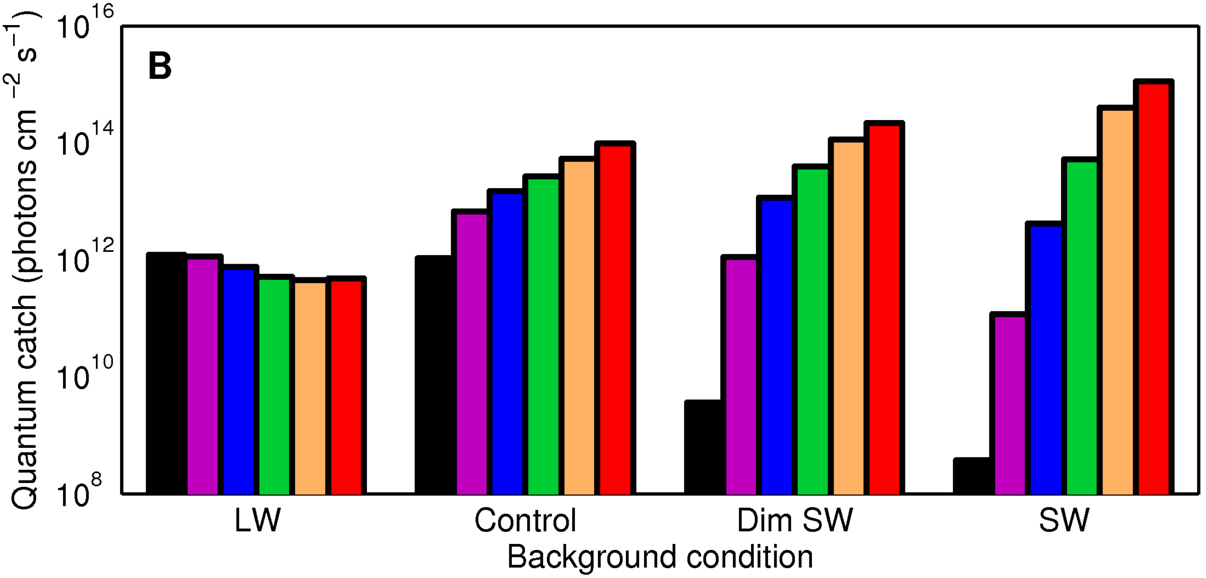

Supplement: Additional file 1 — Background isolation conditions for spectral sensitivity measurements. (a) The spectral irradiance provided under the various background isolation conditions. Background conditions: long-wavelength isolation (LW, red), control (green), Dim short wavelength (Dim SW, cyan) and short-wavelength isolation (SW, blue). (b) The quantum catches of the six possible cone mechanisms under each condition. Cone pigment: SWS1 (black), SWS2b (violet), SWS2a (blue), RH2b (green), RH2a (orange) and LWS (red). Cone quantum catches were calculated while setting the A2% of cones to 50 for the design of the background conditions. [file 1741-7007-8-133-S1.PDF]
