# Supplementary material for: Functional diversity in the color vision of cichlid fishes
Source: BMC Biol. 2010 Oct 28;8:133. doi: 10.1186/1741-7007-8-133 (PMC2988715; doi:10.1186/1741-7007-8-133)
Supplement: Additional file 2 — Summary of t-test results for the examination of the effect of sex on opsin gene expression. [file 1741-7007-8-133-S2.PDF]

Additional file 2 – Summary of t-test results for the examination of the effect of sex on opsin gene expression

	M. auratus			*P. taeniolatus*		
	t	df	p	t	df	p
SWS1	1.536	8	0.1630	0.549	8	0.5983
SWS2b	1.929	8	0.0898	0.316	8	0.7600
SWS2a	1.132	8	0.2904	-1.306	8	0.2278
RH2a	-3.845	8	**0.0049**	-0.646	8	0.5362
RH2b	6.756	8	**0.0001**	1.404	8	0.1980
LWS	0.850	8	0.4200	0.819	8	0.4363

Values in bold indicate significant sex differences in expression. An experiment-wise error rate of 5% was corrected to 0.83% ($\alpha = 0.05/6 = 0.0083$) following Bonferroni correction for 6 hypothesis tests that correspond to 6 opsin genes (RH2aα and RH2aβ were pooled because of genetic and functional similarity).
