# Supplementary material for: Functional diversity in the color vision of cichlid fishes
Source: BMC Biol. 2010 Oct 28;8:133. doi: 10.1186/1741-7007-8-133 (PMC2988715; doi:10.1186/1741-7007-8-133)
Supplement: Additional file 3 — Primer specifications for cone opsin genes. [file 1741-7007-8-133-S3.PDF]

## Additional file 3 - Primer specifications for cone opsin genes

| Target | Primer | Sequence [a] | Primer [b] (bp) | GC (%) | Tm [c] (ºC) | Annealing site | Product [b] (bp) | Location | Input PCR template[d] |
|---|---|---|---|---|---|---|---|---|---|
| *SWS1* | UV F3 | ACATCCCTGAAAG TCTGGGC | 20 | 60 | 55.59 | 655-674 | 148 | Exon III | AF191222 |
| | UV R2 | AGCAGCTGGGAG TAGCAGAA | 20 | 55 | 54.96 | 802-783 | 148 | | |
| *SWS2b* | Blue2 F2b | GCTTGTGGTCTCT TGCTGTGG | 21 | 57 | 55.96 | 437-457 | 151 | Exon I | AF317674 |
| | Blue2 R2b | CCAAACAGAGGT GGAAGTGC | 20 | 55 | 53.29 | 587-568 | 151 | | |
| *SWS2a* | Blue1 F1a | GCAGAGAGGGAA GTGACCAG | 20 | 60 | 53.94 | 1267-1286 | 156 | Exon IV | AF247114 |
| | Blue1 R1a | AGCCTTTGAGAAA CAGGACG | 20 | 50 | 52.35 | 1422-1403 | 156 | | |
| *RH2b* | PaG-Rh2b-F12 | GCCTTGTCATTAC TGGATTC | 20 | 45 | 48.37 | 165-184 | 357 | Exon I-II | DQ088645 |
| | PaG-Rh2b-R2 | AGCAAGAACCACA AGAGACC | 20 | 50 | 51.93 | 521-502 | 357 | | |
| *RH2a* | Green1 F4 | CTGAGAAGGAAGT GACCCGT | 20 | 55 | 53.2 | 1160-1179 | 187 | Exon IV | AF247122 |
| | Green1 R3 | AGCACGTAGATAA CAGGGTTGT | 22 | 45 | 53.51 | 1346-1325 | 187 | | |
| *LWS* | Red F1a | TGAGGGTCCCAAT TACCA | 18 | 50 | 48.57 | 853-870 | 271 | Exon I | AF247126 |
| | Red R0 | GCCCTCAAAGATA CACATTGG | 21 | 48 | 51.15 | 1123-1103 | 271 | | |

[a] Tails associated with restriction sites were removed from the original primer sequences [8,15] with an exception of the *RH2b* primer sequence that was designed in our lab.
[b] Length in base pairs.
[c] Melting temperature.
[d] Accession numbers of gene sequences used to assess the specificity of primers.
