# Supplementary material for: Functional diversity in the color vision of cichlid fishes
Source: BMC Biol. 2010 Oct 28;8:133. doi: 10.1186/1741-7007-8-133 (PMC2988715; doi:10.1186/1741-7007-8-133)
Supplement: Additional file 4 — Statistics of cone interactions for the most frequent cone pigment subset of each species. [file 1741-7007-8-133-S4.PDF]

Additional file 4 - Statistics of cone interactions for the most frequent cone pigment subset of each species

	Background [a]	Spectral range [b] (nm)	Cone weight coefficients						R^{2} [c]
			K_{SWS1}	K_{SWS2b}	K_{SWS2a}	K_{RH2b}	K_{RH2a}	K_{LWS}	
M. zebra	**Control**	340-400	0.919	-	-1.038	-	-	-	**0.862**
**Subset #2**		400-480	-0.173	-	0.848	-0.056	-	-	
		480-540	-	-	-0.979	1.906	-0.449	-	
		540-620	-	-	-	-0.980	1.033	-	
	**LW**	340-400	0.371	-	-0.435	-	-	-	**0.906**
		420-460	-0.856	-	1.313	-1.028	-	-	
		460-520	-	-	-0.387	1.523	-0.625	-	
		540-620	-	-	-	-0.959	1.031	-	
	**SW**	340-400	0.320	-	0.202	-	-	-	**0.826**
		420-480	-0.709	-	0.888	-0.154	-	-	
		480-540	-	-	-0.052	0.734	-0.220	-	
		540-620	-	-	-	-0.194	0.263	-	
M. auratus	**Control**	340-380	1.064	-1.036	-	-	-	-	**0.918**
**Subset #3**		400-440	0.079	0.587	-	-	-	-	
		440-480	-	0.075	-	0.867	-	-	
		480-540	-	-	-	0.325	0.735	-	
		540-640	-	-	-	0.565	-	0.776	
	**LW**	340-400	0.584	-0.057	-	-	-	-	**0.883**
		420-460	-5.225	0.589	-	-	-	-	
		480-520	-	-1.835	-	0.915	-	-	
		520-560	-	-	-	-0.800	1.225	-	
		560-640	-	-	-	-1.894	-	0.860	
	**SW**	340-420	0.487	0.492	-	-	-	-	**0.983**
		420-460	-3.387	0.770	-	-	-	-	
		460-520	-	-0.801	-	1.042	-	-	
		520-580	-	-	-	-0.241	0.666	-	
		580-640	-	-	-	2.728	-	0.224	
P. taeniolatus	**Control**	380-420	-	1.321	-	-2.955	-	-	**0.960**
**Subset #7**		440-520	-	-0.945	1.099	0.341	-	-	
		520-580	-	-	-	-	0.153	0.563	
		580-660	-	-	-	-3.260	-	0.803	
	**Dim-SW**	380-420	-	1.327	-	-2.590	-	-	**0.955**
		420-460	-	-	0.897	-0.267	-	-	
		460-520	-	-0.149	-0.068	1.010	-	-	
		520-560	-	-	-	-	0.730	-0.140	
		560-660	-	-	-	-0.536	-	0.570	
	**SW**	380-420	-	2.047	-	-4.080	-	-	**0.988**
		420-460	-	0	2.473	-2.427	-	-	
		460-540	-	-0.199	-0.459	1.294	-	-	
		540-580	-	-	-	-	0.243	0.285	
		560-660	-	-	-	1.053	-	0.329	

[a] Background conditions used for spectral sensitivity measurements. Artificial conditions: LW - Long wavelength isolation, Control, SW - Short wavelength isolation, Dim-SW - Dim short wavelength isolation. Natural conditions: 5 m, 10 m, and 15 m depth.
[b] Modeled spectral range.
[c] Total amount of variance accounted for by the model across the spectrum.
